# Total knee arthroplasty using a hybrid navigation technique

**DOI:** 10.1186/1749-799X-6-26

**Published:** 2011-05-26

**Authors:** Alvin Ong, Kwang Am Jung, Fabio Orozco, Lawrence Delasotta, Dong Won Lee

**Affiliations:** 1 Department of Orthopaedic Surgery, Rothman Institute, New Jersey, USA; 2 Joint and Arthritis Research, Department of Orthopaedic Surgery, Himchan Hospital, 404-3, Mok-dong, Yangcheon-gu, 158-806, Seoul, South Korea; 3 The Webb School of California, CA, USA

## Abstract

The use of computer navigation is becoming a well-recognized technical alternative to conventional total knee arthroplasty (TKA). However, computer navigation has a substantial learning curve and the use of commercially available navigation systems increases surgical time. In addition, the potential risks associated with the navigation TKA, such as, registration errors, notching of the anterior femoral cortex, oversizing of the femoral component, and overresection must be taken into consideration. On the other hand, conventional techniques are familiar and intuitive to most practicing surgeons, and thus, are easier to perform and are less prone to anterior notching and femoral component oversizing. However, conventional techniques have greater risks of inaccurate and inconsistent component alignment than computer navigation. This paper describes a novel technique that combines computer navigation and conventional TKA.

## Introduction

The use of computer navigation for primary total knee arthroplasty (TKA) provides the benefits of accurate bone resection, low outlier frequencies, and the restoration of overall mechanical alignment. However, its use also involves the disadvantage of change in technique and workflow that have been associated with steep learning curve and increased surgical time. Furthermore, several investigators have described the potential risks associated with the use of navigation, which include registration errors, notching of the anterior femoral cortex, oversizing of the femoral component, and overresection [1-4]. These risks mean that surgical plans provided by navigation software might require modification intra-operatively, based on the surgeon's experience and knowledge. On the other hand, conventional TKA has the advantages of familiarity and simplicity. Furthermore, decisions regarding bony resection level are based on measurements taken using a traditional jig and rod, and thus, anterior notching and femoral component oversizing can be avoided. Unfortunately, the conventional technique is more inaccurate and inconsistent in terms of its component alignment ability than computer navigation [5,6]. In this paper, we describe a hybrid technique that combines the benefits of computer navigation and conventional TKA. This hybrid navigation technique was developed to allow TKA to be performed in-line with accepted conventional TKA practice, but with the accuracy of computer navigation.

## Methods

### Indications & Contraindications

The devised hybrid navigation technique was indicated for all 3500 knees that underwent TKA at our institute between Jan 2007 and April 2010. In no case was the hybrid navigation technique deemed to be contraindicated, and the procedure was not aborted intraoperatively in any case. With regard to contraindication, in theory, hardware in the distal femoral metaphysis and diaphysis that might interfere with intramedullary rod placement would pose the only potential contraindication to the use of the technique.

### Preoperative Planning

No special preoperative planning was performed before hybrid navigation. In our practice, we routinely obtain standing anteroposterior (AP), posteroanterior (PA) and lateral radiographs for all patients scheduled for TKA. These images provide an overall picture of deformities present and of the corrections necessary. In addition, they provide information on the presence of hardware, extra-articular deformity, and bone loss. The goal of surgery is to achieve a final mechanical axis of 0 degrees, but we accept up to 3 degrees of overall varus or valgus malalignment.

### Surgical Steps

**Figure 1:**
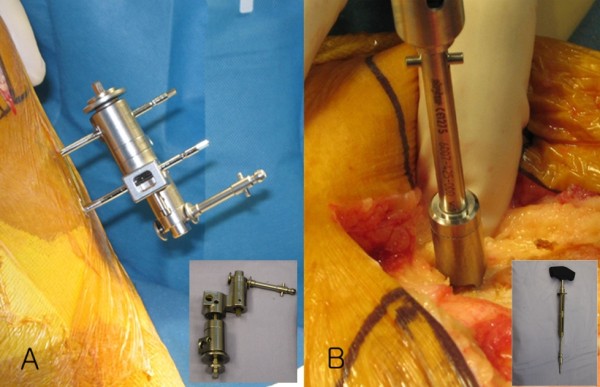
**Two 3 mm Apex pins (A) were positioned in the proximal tibia 10 cm below the tibial joint and a single anti-rotation pin (B) was placed off center in the metaphysis approximately 4 cm above the trochlear articular surface**.

**Figure 2:**
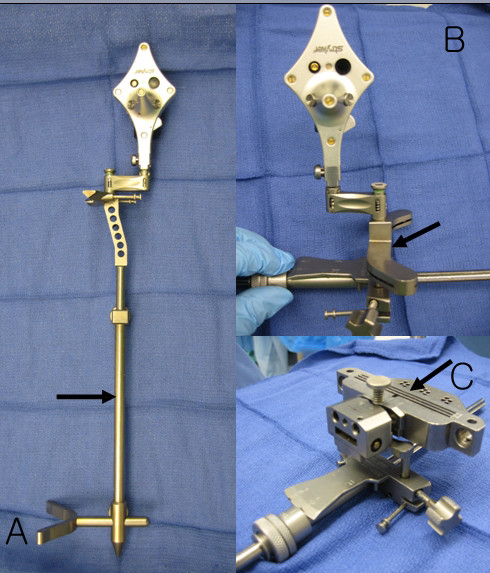
**Standard extramedullary tibial cutting guide (A, arrow), intramedullary distal femur alignment guide with a femoral rotation cutting guide (B, arrow), and a distal femoral cutting block (C, arrow) were modified to accommodate a navigation tracker**.

**Figure 3:**
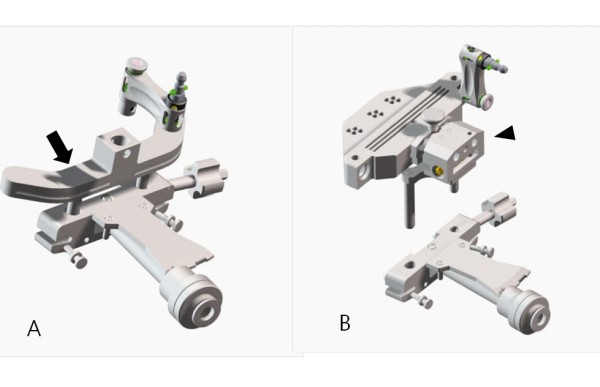
**Navigation enhanced femoral rotation cutting guide (arrow) and a navigation enhanced conventional distal alignment guide with a distal femoral resection pivotal cutting block (arrowhead) were attached to the conventional distal alignment guide as shown (A,B)**.

**Figure 4:**
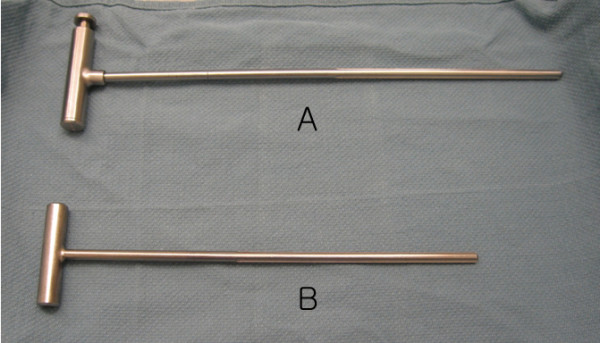
**Conventional femoral intramedullary rods (A) were shortened by 25 cm (B)**.

**Figure 5:**
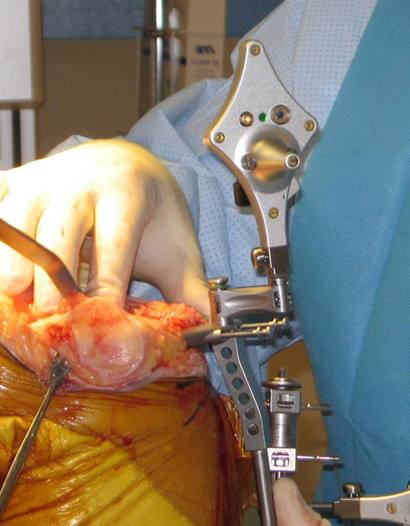
**The modified conventional tibial guide with a tracker was first fixed to the tibia**. Resection height and tibial slope were controlled manually under navigation guidance.

**Figure 6:**
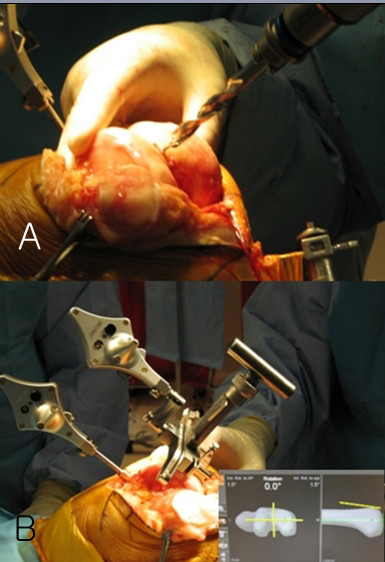
**A "starting" hole was created in the distal femur for IM rod insertion (A)**. The femoral component rotational axis was controlled under navigation guidance using a tracker connected to the anterior femoral cutting jig (B).

**Figure 7:**
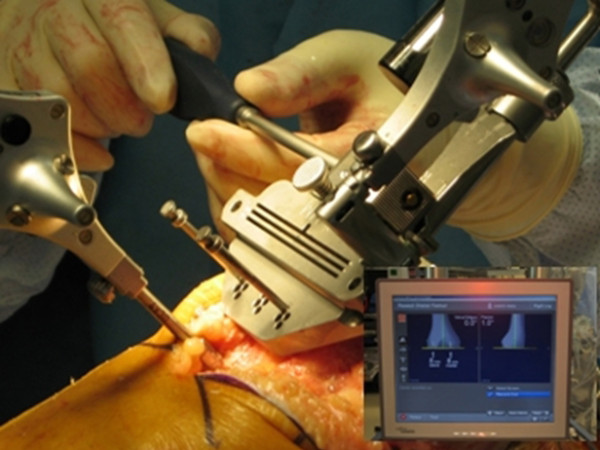
**Resection level and its precise position were controlled and "fine-tuned" using a screwdriver to distal femoral resection pivotal cutting block**.

**Figure 8:**
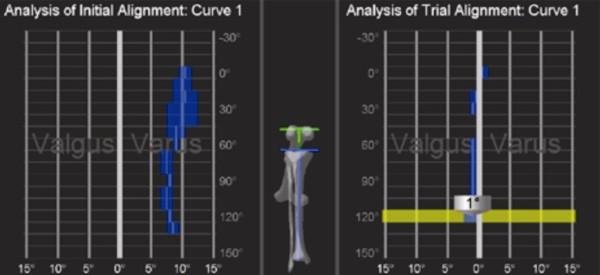
**After trial reduction, tibio-femoral mechanical alignment was recorded and compared with preoperative tibio-femoral mechanical alignment**.

The Stryker image-free knee navigation system (Stryker Navigation, Kalamazoo, Michigan, USA) was used in all cases; however, any commercially available navigation system can be modified for use with the hybrid technique (described below.) All patients received a posterior-stabilized knee system, and all patellae were resurfaced. The implants used were the Triathlon implant (Stryker; Mahwah, NJ, USA) and the Genesis II total knee implant (Smith & Nephew; Memphis, USA). A medial parapatellar approach and an anterior-referencing technique were used in all cases, and all implants were cemented. The navigation computer is best positioned opposite the surgeon approximately 4 feet away from the patient. The camera is located over the patient's knee and directed downward at 45 degrees. Prior to exsanguination of the limb and incision, navigation trackers (light emitting diode) were fixed to both the distal femur and proximal tibia. Two 3 mm Apex pins were utilized on the distal femoral metaphysis and proximal tibial metaphysis in conjunction with the Stryker OrthoLock System (Stryker, Kalamazoo, Michigan, USA) (Figure 1). We recommend that these pin clusters be placed approximately 10 cm distal to the joint line in the proximal tibia, such that they do not interfere with the surgical incision or the operative field. Likewise, we recommend that pin clusters be placed approximately 10-15 cm proximal to the joint line in the distal femur, such that they do not interfere with the trajectory of the intramedullary rod. We do not recommend placement of pins in the diaphysis, due to the risks of thermal necrosis and stress fracture. Furthermore, we recommend that the pins be placed in different planes to avoid the creation of a stress riser in bone; alternatively, a single pin technique can be utilized using a Stryker Anti-rotation pin (Stryker, Kalamazoo, Michigan, USA) (Figure 1). One pin was placed in the metaphysis either medial to or lateral to midline (beyond the trajectory of the intramedullary rod.) Care must be taken to ensure that the femoral and tibial trackers are positioned in direct view of the navigation camera. In all cases, a standard extramedullary tibial cutting guide, an intramedullary distal femur alignment guide, a femoral rotation cutting guide, and a navigation-enhanced distal femoral cutting block (Stryker, Mahwah, NJ., USA)(Figure 2, 3) were utilized; each of these instruments was modified to allow them to accommodate a navigation tracker. A tracker was attached to navigation enhanced femoral rotation cutting guide and navigation enhanced conventional distal alignment guide with distal femoral resection pivotal cutting block (Figure 2,3) The conventional femoral intramedullary rod (Figure 4) was shortened by 25 cm to avoid interference with the tracker pin on the femoral side. In terms of surgical steps, the centers of the femoral head, knee joint, and ankle joint were identified, and then surface mapping of anatomic landmarks of the knee was performed. After the anatomical survey, navigation of the femoral and tibial bone resection was performed using Stryker software (eNact Knee Navigation Software 3.1). The navigation system had axis and alignment incremental changes of 0.5 degree and the resection level and height in millimeter increments. The modified conventional tibial guide with a tracker was first fixed to the tibia; resection height and tibial slope were controlled manually under navigation guidance (Figure 5). After completing the tibial resection, a "starting" hole was created in the distal femur for IM rod insertion (Figure 6). This "starting" hole was made just above the notch centered between the lateral and medial condyle. A modified short IM rod with a conventional distal alignment guide and tracker was then inserted into the opening. The femoral component rotational axis was controlled under navigation guidance using a tracker connected to the anterior femoral cutting jig (Figure 6). Rotation is based off the transepicondylar axis. After determining femoral component rotation, an anterior rough cut was performed using the conventional jig-based technique. Subsequently, the distal femoral resection pivotal cutting block was connected to the conventional distal femur alignment guide. The resection level and the exact position of distal femoral resection were controlled and "fine-tuned" using a screwdriver (Figure 7). Flexion of the distal femur was set at approximately 3-5 degrees using the IM rod to accommodate femoral bow. After performing the distal femoral cut, the anterior/posterior and chamfer cuts were completed using a selected system-specific 6-in-1 or 4-in-1 femoral cutting block. Depending on the balance of flexion and extension gaps, minimal bone adjustment was carried out under navigation guidance. After trial reduction, tibio-femoral mechanical alignment in knee extension and flexion were recorded and their kinematic curves were compared with preoperative tibio-femoral mechanical alignment. (Figure 8) After every surgical step, the accuracies of bone cuts were assessed with the aid of the navigation system and a resection plane probe. Cuts were corrected as necessary if they were deemed to be outside the acceptable range. After confirming their accuracies and soft tissue balance, real components were implanted with cement using the standard technique.

## Brief Results

More than 3500 knees underwent primary total knee replacement from Jan 2007 to April 2010. The first 50 knees treated (mean age 65.2 years) and the last 50 knees treated (mean age 64.3 years) were compared with respect to surgical time and component alignment to assess the effects of the learning process. Coronal and sagittal alignments of femoral components for the first 50 knees were mean valgus 0.5°and mean flexion 3.5° and these values were similar for the last 50 knees (mean valgus 0.2° and mean flexion 3.6°). For tibial components of the first 50 knees, mean coronal and sagittal alignments were valgus 0. 3° and flexion 2.5°, and these were also similar for the last 50 knees (mean valgus 0.3° and mean flexion 2.7°). Overall mechanical alignments for the first and last knee groups were mean varus 1.5° and 1°, respectively, and mean operation times (skin incision to skin closure) were 61 and 50 minutes, respectively. There were three cases of tibial fracture attributed to a tracker pin, but these fractures were considered to be related to general concerns of navigation TKR, and not to a system-specific problem. Ten cases developed a superficial infection at a tracker pin site, but no case of fat embolism occurred.

## Discussion

Computer navigation is becoming a well-recognized technical alternative to conventional total knee replacement, but its merits and demerits continue to be widely debated [7-11]. Computer navigation has the disadvantages of a protracted learning curve and increased surgical time [11] In addition, several investigators have suggested that navigation might increase the risks of notching of the anterior femoral cortex and oversizing of the femoral component. In particular, Minoda et al. [3] found that 40-85% male cases and 65-100% of elderly female cases treated with navigation showed anterior notching. Matsumoto et al. [2] suggested that surgeons should be aware of the potential for oversizing when determining the size of the femoral component, particularly when the femoral bone is anteriorly bowed. Kim et al. [10] also reported a higher incidence of anterior femoral notching in navigation treated knees than in conventionally treated knees. However, these problems might be due to discrepancies between the anterior bow of the femur and its straight mechanical. More specifically, computer navigation calculates the sagittal axis of the femur by drawing a straight line between the center of the femoral head and the center of the knee, and thus, femoral bow is not taken into consideration, and therefore, cannot be determined from anatomic registration points. Furthermore, decision making regarding resection level using navigation might be difficult, especially in knees with a deformed articular surface, such as, severe varus or valgus knees, as compared with decision making using the conventional technique. Kim et al. [10] reported overresection of proximal tibial bone as a complication of navigation, and thus, the surgical planning provided by the navigation software might require modification based on surgeon's experience and knowledge of the surgical procedures. The hybrid navigation system described here was devised to combine the ease of use of classic conventional resection instruments and the accuracy of the navigation technique. Furthermore, the use of an intramedullary rod in conjunction with navigation allows femoral bow to be taken into consideration. In theory and practice, the rod is deflected by femoral bow, which allows flexion of the femoral component to accommodate femoral bow, which facilitates appropriate flexion of the femoral component and prevents inadvertent notching of the anterior femoral cortex. This use of an intramedullary rod in conjunction with navigation represents an advantage of the hybrid technique over the pure navigation technique wherein femoral bow is not taken in account when determining femoral component position.

Although it has been reported that the use of a femoral intramedullary IM rod might increase the possibility of a fat embolism [12,13], it appears that the use of a smaller diameter, shorter IM rod may reduce this risk. On the other hand, Kim et al. [14] found that the use of an IM rod did not increase the risk of fat embolism or increase perioperative blood loss.

The present study shows that the hybrid navigation technique increases the accuracy of component alignment versus the conventional technique and requires less time than navigation technique. Furthermore, our findings indicate that hybrid technique does not require a protracted learning process. In addition, no case of fat embolism was encountered. Accordingly, we believe that the described hybrid navigation technique enables TKA to be conducted safely and precisely without femoral notching or femoral component oversizing.

## Conclusion

Considering several manufactures' navigation systems with their own successful benefits, We do not present the devised hybrid navigation technique as a definitive method for navigation TKR. Nevertheless, we believe that this technique should be considered as an alternative means of conducting navigation TKR.

## Competing interests

Alvin Ong is a consultant for Stryker Orthopaedics (Mahwah, NJ). All the other authors have no competing interests.

## Authors' contributions

AO and KAJ conceived the project, conducted the primary literature review and drafted the manuscript. FO, LD, and DWL contributed to the literature review, the manuscript preparation, and provided the photographs. All authors read and approved the final manuscript.

## Consent

All authors certify that the human research protocol used during this investigation was approved by our institution and that all investigations conducted during this study conformed with ethical research principles.
